# Wave propagation of cortical population activity under urethane anesthesia is state dependent

**DOI:** 10.1186/1471-2202-14-78

**Published:** 2013-07-31

**Authors:** Tim Wanger, Kentaroh Takagaki (高垣堅太郎), Michael T Lippert, Jürgen Goldschmidt, Frank W Ohl

**Affiliations:** 1 Leibniz-Institute for Neurobiology, Magdeburg, 39118, Germany; 2 Otto-von-Guericke-University, Magdeburg, 39106, Germany; 3 Center for Behavioral Brain Science (CBBS), Magdeburg, Germany

**Keywords:** Traveling wave, Wave propagation, Cortex, State-dependence, Voltage-sensitive dye imaging, Urethane, Current source density, Rat visual cortex

## Abstract

**Background:**

Propagating waves of excitation have been observed extensively in the neocortex, during both spontaneous and sensory-evoked activity, and they play a critical role in spatially organizing information processing. However, the state-dependence of these spatiotemporal propagation patterns is largely unexplored. In this report, we use voltage-sensitive dye imaging in the rat visual cortex to study the propagation of spontaneous population activity in two discrete cortical states induced by urethane anesthesia.

**Results:**

While laminar current source density patterns of spontaneous population events in these two states indicate a considerable degree of similarity in laminar networks, lateral propagation in the more active desynchronized state is approximately 20% faster than in the slower synchronized state. Furthermore, trajectories of wave propagation exhibit a strong anisotropy, but the preferred direction is different depending on cortical state.

**Conclusions:**

Our results show that horizontal wave propagation of spontaneous neural activity is largely dependent on the global activity states of local cortical circuits.

## Background

Spontaneous and sensory-evoked population activity in the mammalian neocortex is known to manifest in the form of traveling or propagating waves (e.g. [1-8]). Among other functions, these waves are postulated to provide nonspecific background depolarization to neuronal assemblies, thus modulating the likelihood of action potential initiation within spatially segregated populations of cells [9,10]. Despite this proposed role of propagating waves in spatially coordinating neuronal computation [11-15], the mechanisms governing wave propagation remain an issue of ongoing debate [16]. In particular, little is known about how propagation dynamics are influenced by global network states [17].

Distinct global network states are a critical feature of neocortical dynamics, and characterize sleep and wakefulness [18], as well as stages of arousal and anesthesia [19]. These global network states can have profound effects on cortical information processing by biasing the dynamics of membrane potential synchrony [20] and the interaction of sensory evoked responses with ongoing activity [21,22]. Thus, changes in cortical state should lead to pronounced changes in the operation of cortical networks underlying wave propagation, but investigations to date have mainly focused on wave propagation in single states [17]. Therefore, in order to comprehensively account for the mechanisms of cortical wave propagation and to understand differences in observations from different experimental and clinical preparations, insight into the state-dependence of propagation patterns is crucial.

**Figure 1:**
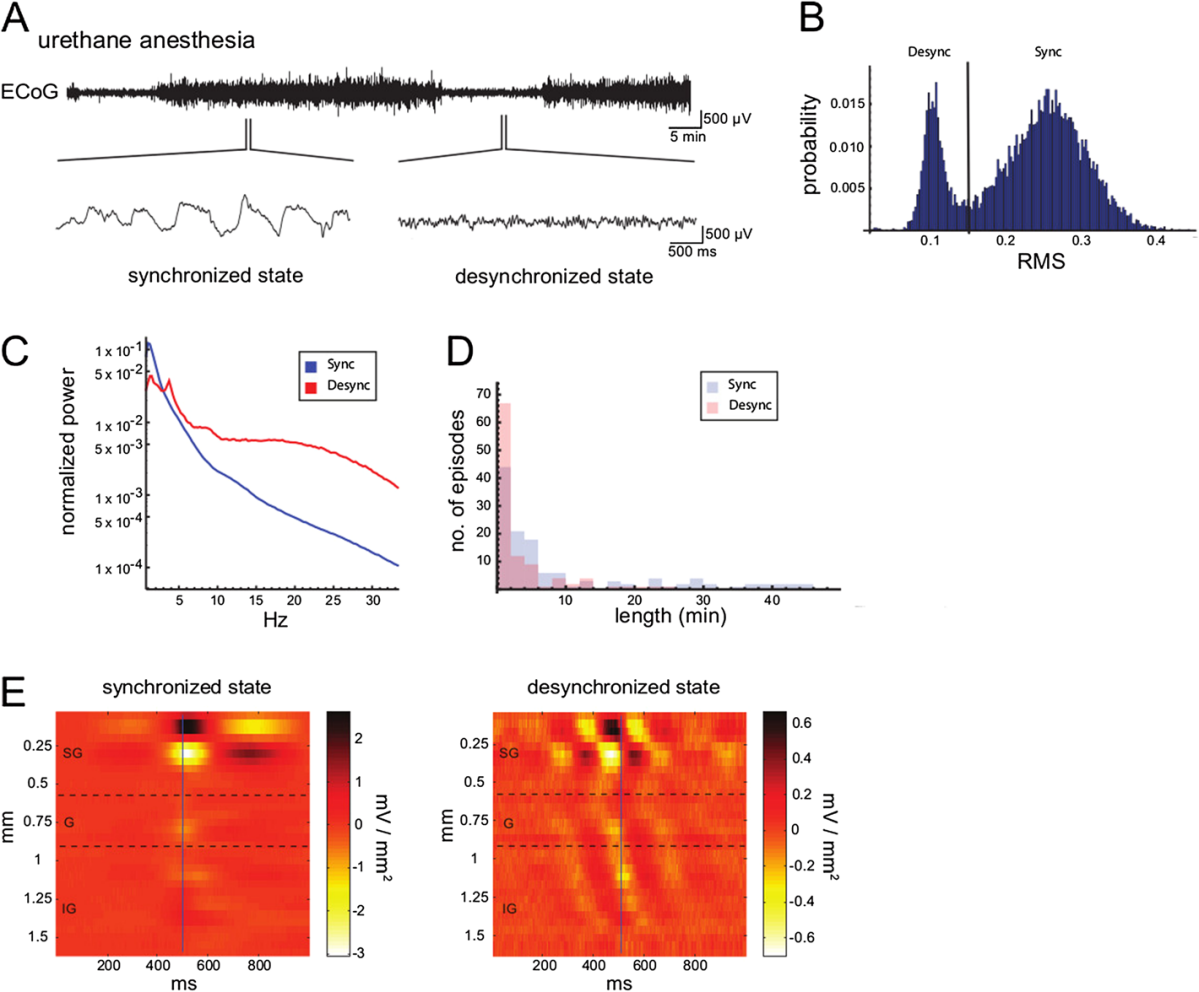
**Properties of spontaneous population activity in rat visual cortex. (A)** Spontaneous state alternations of the ECoG between ECoG-synchronized and ECoG-desynchronized states under urethane anesthesia. During synchronized states, the ECoG exhibits a large-amplitude slow-wave pattern, whereas during desynchronized states the ECoG shows a low-amplitude fast-wave pattern. **(B)** Bimodal distribution of ECoG amplitude RMS values under urethane anesthesia. Data are from ~8 h of spontaneous ECoG recording. **(C)** ECoG power spectrograms of the two urethane states. Note the higher power in the slow frequency range (< 3 Hz) in the synchronized state as compared to the desynchronized state, whereas the reverse holds true for frequencies faster than 3 Hz. Also note a distinct spectral peak at around 4 Hz in the desynchronized state. **(D)** Histogram showing the durations of each state (pooled data from three animals). While on average, the length of each cycle is on the order of < 15 min, episodes of the urethane synchronized state can last up to 50 min, especially during deep anesthesia. **(E)** Averaged CSD plots along the depth of the cortex, triggered off of spontaneous activity patterns (see Methods). Note that despite differences in amplitude, laminar CSD profiles are qualitatively similar across states, with net densities of current flow being highest in supragranular (SG) layers (Sync: AVREC_SG_/AVREC_total_ = 0.684 ± 0.044; Desync: AVREC_SG_/AVREC_total_ = 0.698 ± 0.022, ± SEM). Furthermore, no prominent granular (G) or infragranular (IG) sink-source pairs are apparent in profiles of either state. These findings suggest that spontaneous population activity is, to a large degree, governed by similar anatomical network components in synchronized and desynchronized states.

Here, we address the state-dependence of lateral intracortical networks by analyzing spatiotemporal patterns of propagating population activity as recorded with voltage-sensitive dye (VSD) imaging in rat visual cortex, under a high-amplitude ECoG-synchronized state and a low-amplitude ECoG-desynchronized state induced by urethane anesthesia. Under moderate planes of urethane anesthesia, the ECoG (electrocorticogram) spontaneously alternates between these two states (Figure 1A), which have been shown to resemble, in some respects, forebrain states observed during sleep [23,24]. We first used laminar current source density (CSD) analysis to investigate the cortical laminar organization of the spontaneous population activity patterns in each state. Using a temporospatial flow detection algorithm [25], we then quantified local propagation patterns of spontaneous activity in single trials. Our results show that, despite a large degree of similarity in laminar networks, propagation of spontaneous excitable waves is very susceptible to cortical state.

## Methods

All experiments were in compliance with the guidelines of the European Community (EUVD 86/609/EEC) and were approved by an ethics commission of the state of Sachsen-Anhalt. Twelve adult male Wistar rats (250–400 g) were used for the experiments described here.

### Anesthesia

Induction of anesthesia was performed by either intraperitoneal or intravenous infusion of an aqueous solution of urethane (1.25–1.5 g/kg). Further anesthetic was given as necessary to maintain areflexia.

### CSD analysis

To analyze the layered activity distribution in both cortical states, three animals were implanted with a silicon microelectrode (32 recording sites spaced 50 μm apart, each 400 μm^2^, NeuroNexus Technologies, Inc.) into the primary visual cortex (V1). The back surface of the electrode was coated with the fluorescent carbocyanine dye DiIC18 to later localize the recording site in histological slices [26]. Laminar positioning of the electrode was controlled for by inserting it 1.6 mm below the brain surface. Cortical layers were delineated based both on histology and on the typical laminar CSD patterns following visual stimulation [26]. Electric field potentials were recorded with a biosignal recording system (MAP System, Plexon Inc.) and split into local field potentials (LFP, 1–150 Hz) and multi-unit activity (MUA, 0.9–8.8 kHz). LFPs were digitized continuously, and triggered spike data were saved as waveforms and timestamps for offline analysis.

CSDs were obtained from LFP signals as described previously [26,27]. The calculation of the characteristic CSD profile for both states was done within a window of 1000 ms. In the synchronized state, which continuously cycles between phases of generalized network activity and network silence [28], the window was centered on each time point where the integrated MUA (binned at 5 ms) crossed a threshold of three standard deviations above baseline (i.e. the preceding bin window) and remained higher for at least 500 ms. We found this triggering to be considerably more accurate than triggering off of the low-pass filtered LFP. For the desynchronized state, thresholding based on MUA cannot be used, since neurons are constantly discharging [22]. We therefore filtered the LFP in a band of 3–6 Hz, which typically shows a spectral peak in the desynchronized state (Figure 1C). After band-pass filtering, we calculated the Hilbert-phase of the signal and triggered the average calculation at zero degree phase angle. This procedure results in both cases in an average CSD profile of thousands of events, locked to either down-up state transitions or phase crossings. Averaged rectified CSDs (AVRECs, [27] were calculated from 200 ms windows around the trigger.

### VSD imaging

For VSD imaging, methods were adapted from our previous work [3,29]. Briefly, craniotomy of approximately 5 mm diameter was performed over the visual cortex of the left hemisphere in nine animals (center: bregma: 7 mm, lateral: 4 mm) and cortex was stained transdurally with the dye RH-1691 (1 mg/ml in normal saline; Optical Imaging) for 1.5 h. Optical signals were recorded and processed as described previously [3,29].

ECoGs were recorded simultaneously with a 1 mm Ag/AgCl ball electrode placed on the edge of the exposed cortical surface in the lower left corner of the imaging field (plan view, rostrocaudal axis). A second Ag/AgCl electrode placed over the cerebellum served as reference. Signals were amplified with a custom-made 1000-gain amplifier and filtered in a band of 0.5–100 Hz, prior to digitization. The electrocardiogram was recorded as well, to allow triggered removal of heartbeat-related artifacts from the optical recording [3].

### Classification of cortical state

In order to perform offline classification of cortical states, root mean square (RMS) values of 2 s segments were determined from filtered LFP or ECoG recordings. The resulting bimodal distribution was then divided at the saddle point to classify the segment into distinct brain states (Figure 1B). Segments with an RMS value larger than criterion were classified as synchronized state, whereas segments with values smaller than criterion were classified as desynchronized state. Power-spectral densities were estimated from 2 s long, Hamming-windowed segments, and normalized by the integral of power over frequency within the specified window. Data were processed in Matlab (Mathworks) and Mathematica (Wolfram Research).

On average, episodes of each cortical state lasted between 2–10 min (Figure 1D). However, some episodes of the synchronized state lasted up to 50 min (Figure 1D). Such persistent episodes of the urethane synchronized state were found to be particularly prevalent during the first few hours of each recording session, when anesthesia levels are presumably at their deepest. At later stages in the experiment, alternations between synchronized and desynchronized episodes became more frequent and the average duration of each discrete state shortened (data not shown).

### Quantification of propagation patterns

In order to characterize the lateral propagation velocity and trajectories of spontaneous activity in VSD recordings, we applied a temporospatial flow detection algorithm [25]. Briefly, this algorithm compares signal traces from neighboring detectors and extracts the time shift necessary to obtain best correlation coefficients between signal traces. The algorithm then aggregates the spatial pattern of these time shifts from neighboring detector pairs, to obtain the propagation pattern within the local area. Although this algorithm can detect the three known patterns of spatial propagation [10], translation waves, source waves and spiral waves, in our experiments, translation waves were predominant and therefore the other patterns were not analyzed in further detail. Software to perform these calculations is freely available from (http://www.sourceforge.net/projects/nounou). The performance characteristics and implementation details of this algorithm are discussed in detail elsewhere [25].

**Figure 2:**
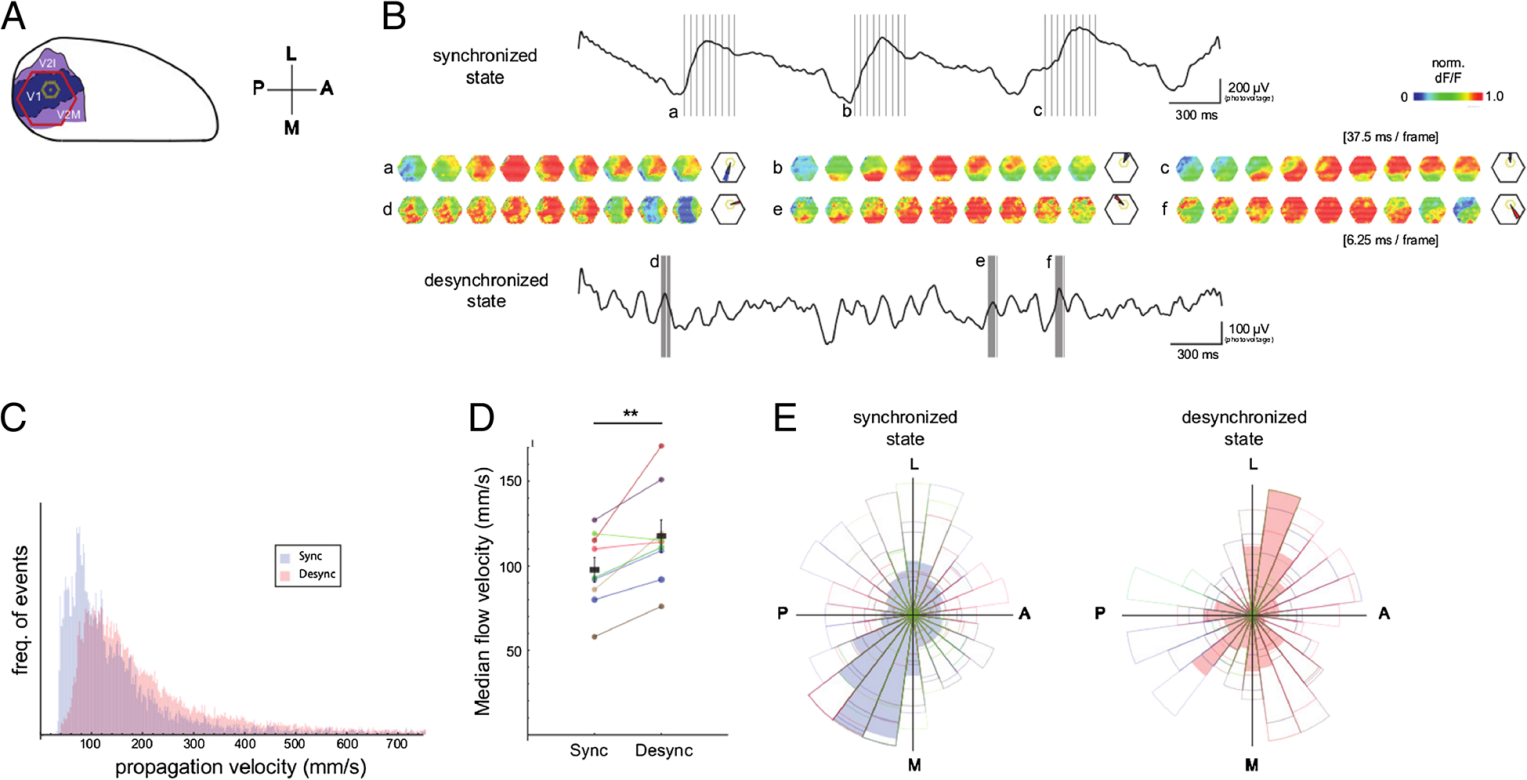
**State-dependence of spontaneous cortical propagation. (A)** Imaging field with a 464-channel photodiode array covering V1 and V2M. Selected detectors within V1 are highlighted in yellow. **(B)** Representative single-trial examples of spontaneous cortical waves obtained by VSD imaging. Vertical lines and lower-case labels indicate time periods for which frames are drawn. Inset frames: frames show propagation of activity within the imaging field (normalized scale, variable scaling). Note that in the desynchronized state, propagation patterns are more spatially fragmented as compared to the synchronized state. Black contour frames: Rose plots indicating flow trajectories for each example wave a-f as obtained by the temporospatial correlation algorithm described below (arbitrary scaling). **(C)** Local velocity of lateral propagation of spontaneous cortical waves as obtained by a temporospatial correlation algorithm applied to a hexagonal ring of detectors as indicated in **A**. Data from a representative animal are shown. Note that spontaneous waves in the desynchronized state tend to propagate faster than spontaneous waves in the synchronized state. **(D)** Statistical evaluation of the state-dependence of propagation velocity indicated in **C**. Medians of the respective non-Gaussian distributions from nine animals are shown (circles). Different colors indicate different animals. Means of the medians are indicated by black rectangles (± SEM). Matched-pairs signed-ranks test (**p < 0.02). **(E)** Rose histograms showing propagation preferences of spontaneous cortical waves **(**colored outlines, same color coding by animal as in **D)**. Histograms were normalized to the maximum bin count for each state separately. Detectors were the same as in **C**. Note that the average flow histogram (transparent blue and red) indicates highly anisotropic propagation in both states, the axis of which is approximately equivalent in both states. The preferred trajectories of propagation within that axis are clearly state-dependent (Kuiper’s test, *** p < 0.001 for every animal).

In the present study, a correlation window of 200 ms (320 frames) in combination with a maximum shift window of 25 ms (40 frames) was used to calculate temporospatial flow; correlation windows of 150 and 250 ms showed similar results (see Additional file 1). These windows are sufficiently large to encompass the main features of the activity pattern, while not so large as to increase the risk of aliasing across cycles of oscillation. Of note, given the subsequent spatial pattern matching in our flow algorithm, such aliasing would be rejected from the dataset and would therefore lead to lower sensitivity of the algorithm, but would not bias the results. For detection of spontaneous flow events, the algorithm was applied to signal traces from a hexagonal ring of detectors centered in V1 as indicated in Figure 2A. The correlation window was moved in successive steps of 4 frames over the bandpass-filtered (0.5–35 Hz) VSD signal, which consisted of 2 s long traces of spontaneous activity from each detector. Prior to flow detection, VSD traces were classified as synchronized or desynchronized based on the criterion described above. The obtained flow vectors were than matched to templates and detected events with flow reliability lower than 0.85 were discarded [25]. Flow trajectory distributions were tested for statistically significant differences using a circular Kuiper two-sample test from the CircStat toolbox [30].

## Results

We investigated the state-dependence of spontaneous laterally propagating waves in the urethane-anesthetized rat visual cortex. We first examined the laminar organization of these events using CSD analysis of silicon microelectrode recordings to investigate the network population activity associated with each state. We then used VSD imaging to record spontaneous activity in both states, and compared the characteristics of spatiotemporal propagation, i.e. propagation velocities and trajectories.

### Laminar networks of spontaneous population activity are similar for synchronized and desynchronized states

Desynchronized forebrain states are characterized by higher spontaneous firing rates of thalamic neurons than synchronized slow-wave states [31]. Therefore, it could be hypothesized that wave propagation in the desynchronized state may potentially involve a stronger thalamic component, whereas propagation patterns in synchronized states are thought to be mainly governed by intracortical inputs [10,16]. Such a network difference would obscure comparison of propagation patterns across states. As one means to address this concern and to investigate which laminar cortical layers are most involved in these spontaneous activity patterns, we used laminar CSD analysis [32].

For the synchronized state, CSD profiles were obtained by averaging spontaneous events centered on the transition from silent to active network states. For the desynchronized state, averaging was triggered off of the instantaneous phase of its main oscillatory band. In all three animals examined, the resulting CSD profiles were qualitatively similar across states, in terms of the laminar arrangement and the relative amplitudes of respective current sinks and sources. In particular, both states featured characteristic supragranular sink-source dipoles, the amplitude of which was considerably larger compared to respective granular or infragranular components (Figure 1E). The variance of the total AVREC which is explained by supragranular sink-source dipoles was 68.4% (± 4.2%, SEM) in the synchronized state and 69.9% (± 2.2%, SEM) in the desynchronized state, respectively.

Since laminar CSD profiles of synchronized and desynchronized states show no obvious discrepancies, we interpret this as supportive of the proposition that comparison of propagation patterns in these two states is meaningful with respect to the laminar networks involved.

### Horizontal propagation of spontaneous waves is faster in the desynchronized state

Spontaneous propagating waves of depolarization were observed in both cortical states. Waves in the desynchronized state, however, appeared more fragmented spatially (Figure 2B), presumably reflecting the decreased spatial and temporal correlation of membrane potentials in more active forebrain states [20,33].

Local propagation speeds of spontaneous waves varied greatly across events and trials, apparently following Poisson statistics (Figure 2C), with desynchronized probability distributions being significantly biased towards faster speeds. A comparison of the medians of the respective speed distributions from nine animals showed that, on average, propagation speeds were about 20% faster in the desynchronized state as compared to the synchronized state (Figure 2D, mean_SYNC_ = 98 mm/s ± 7 mm/s and mean_DESYNC_ = 118 mm/s ± 9 mm/s (± SEM), Wilcoxon matched-pairs signed-ranks test, p < 0.02).

### Trajectories of spontaneous cortical waves are state-dependent

As for the directional properties of propagation, spontaneous waves measured at the center of V1 traveled in every direction (Figure 2E), but with a highly anisotropic distribution in both states (Figure 2E). While the dominant axis of propagation was always the lateral-medial axis regardless of cortical state, preferred propagation directions within this biased axis were clearly state dependent (Kuiper’s test, p < 0.001 for every animal), differing by 180° for synchronized and desynchronized states. Notably, the respective directions of anisotropy were very stable across animals (Figure 2E), suggesting commonalities in the network organization of these spontaneous waves.

Thus, both the distribution of propagation speeds and of propagation directions showed a clear dependence on the global cortical state.

## Discussion

Based on laminar CSD analyses of the predominant ongoing activity patterns in each state, we first demonstrate that laminar networks in both states demonstrate a considerable degree of similarity. Despite these similarities in network patterns, spontaneous population activity in synchronized and desynchronized states differs, by definition, with respect to its spectral amplitude, as well as in MUA activity patterns [22], cell-type-specific firing patterns [34,35] and membrane potential dynamics [20,36]. In this report, we further demonstrate that this activity also differs in its temporal and spatial characteristics: namely, spontaneous waves of depolarization as recorded by VSD imaging in rat visual cortex tend to propagate significantly faster and with different anisotropies in an activated globally desynchronized state as compared to a globally synchronized slow-wave state.

Waves in synchronized slow-wave states are known to reflect the globalized spread of cellular up states of depolarization across the cortical network [37-39]. In contrast, wave propagation in the urethane-desynchronized state most likely reflects the spread of more localized patterns of oscillatory synchrony (cf. [40]) with a spectral peak around 4 Hz (Figure 1C). Despite these apparent spatiotemporal differences of the propagating waves, laminar CSD profiles of synchronized and desynchronized states appear strikingly similar (Figure 1E).

In particular, the dipolar arrangement of sink-source pairs in supragranular layers, which was observed across states, indicates that the bulk of synaptic transmission is mediated via projections to somata and/or neural processes located in supragranular layers. Mechanistically, the observed supragranular dipoles (cf. [41]) presumably reflect postsynaptic input to somata and dendrites of layer 2/3 pyramidal neurons as well as to the distal dendrites of large layer 5 pyramidal neurons which ramify extensively into superficial layers [42]. After being depolarized to firing threshold, pyramidal neurons in layers 2/3 and 5 would then pass on the excitation to their efferent target neurons via intra- and interlaminar projections [43], resulting in lateral spread of the depolarization as observed in supragranular and infragranular layers of cortical slices [44,45].

Of note, as there is accumulating evidence suggesting a leading role for layer 5 networks in cortical up state initiation [46-50] and intracortical wave propagation [39,45,50], the left panel in Figure 1E should not be misinterpreted as evidence for a supragranular origin of propagating cortical up states. Rather, and within the mechanistic framework described above, we reckon that the prominent supragranular dipoles in Figure 1E are evoked by postsynaptic input coming from infragranular neurons which project onto somata and neural processes in layer 2/3. This scenario is supported by a recent study showing that layer 5A provides strong driving input to supragranular layers [45].

Regardless of exact circuit mechanisms, our laminar CSD patterns do argue against a fundamental functional network difference in the two states, as far as the relative degree of thalamic contribution to the respective current sinks and sources is concerned. For instance, if the contribution of thalamic components to spontaneous population activity would be significantly stronger in the desynchronized state as compared to the synchronized state, one would expect the right panel in Figure 1E to more closely resemble the classical CSD pattern of a prominent granular sink followed by activation of other cortical layers [27,32,49]. However, the question whether lateral propagation of spontaneous waves of excitation is predominantly governed by intracortical mechanisms [15,50-52] or whether there is an essential thalamic component involved [53-55] remains to be answered.

Lateral or horizontal spread of mass depolarization is thought of as propagation along a network of interconnected neurons or as a consequence of the phase delay between coupled local oscillators [9]. In either case, the main factor determining the velocity of propagation is the strength of excitatory-excitatory connections in the network and the proximity of the membrane potential of excitatory neurons to threshold [56]. The difference in lateral propagation velocity of spontaneous cortical waves between the two states can thus be interpreted as an indicator of stronger population synaptic strength, i.e. lateral coupling, in cortical networks under desynchronized conditions. Such changes in lateral coupling may result from the fact that during desynchronized states, the membrane potential of neurons is more depolarized [28], so that incoming excitatory postsynaptic potentials bring neurons faster to threshold as compared to more synchronized slow-wave states, where neurons are more hyperpolarized [28]. This could lead to faster spread of activation across the network. Reported velocities for lateral propagation in the visual cortex differ greatly [16], and our results demonstrate that differences in brain state can contribute considerably to this discrepancy. With respect to cortical information processing, a faster spread of depolarizing waves would facilitate communication between spatially segregated neural ensembles by depolarizing more neurons in a shorter time window, thus enhancing the gain for incoming stimuli in a larger patch of cortical tissue. This, in turn, might be a factor contributing to efficient integration of information in desynchronized forebrain states [57] which accompany attentive modes of processing like dreaming or wakefulness.

Of note, sensory-evoked cortical waves propagate about twice as fast as spontaneous waves in the same cortical state (see Additional file 1), and propagation parameters of evoked waves are not significantly affected by the global cortical state (Additional file 1). These pronounced differences in propagation velocity between spontaneous and sensory-evoked waves are readily explained by the qualitatively different nature of spontaneous and evoked waves in our study. Since our visual stimulus is not a point-source (which is impractical in rodent visual cortex), the spread of these sensory-evoked waves is more strongly governed by subcortical-cortical interactions [10]. Mechanistically, in this case, a patterned feedforward input from the thalamus might excite neighboring cortical regions with a time delay, the result of which would be an apparent wave motion pattern, corresponding to a subcortical oscillator model where a phase delay in excitatory drive results in fictive wave motion [9]. To test this proposition requires experiments in a sensory area which can be stimulated with a point-source, such as the barrel cortex [58].

Since wave propagation is thought to have functional relevance in spatial integration, the state-dependence of preferred propagation trajectories is of particular note. Whereas anisotropic propagation of spontaneous cortical waves has been observed in both humans [2,5] and animals [12,15,59], a brain state-dependent change in the directions of spontaneous traveling waves has, to our knowledge, not been demonstrated to date. Given that preferred trajectories associated with each state were fairly stable across animals, our data suggests a strong systematic modulation of effective network pathways by cortical state. A reversal in the bias of the direction of information transfer between higher- and lower-order visual areas [11] could potentially associate with different modes of "top-down" or "bottom-up" processing, and an important future direction will be to test the behavioral effects of selective inhibition of directional propagation.

## Conclusions

While lateral propagation may depend on local cortical circuits [40], our results demonstrate that the global activity state of the cortical network also has a significant impact on the velocity and preferred direction of propagating waves. Our findings shed light on how propagation parameters, and thus spatiotemporal integration, changes as a function of cortical state.

## Abbreviations

AVREC: Averaged rectified CSD; CSD: Current source density; ECoG: Electrocorticogram; LFP: Local field potential; MUA: Multi-unit activity; RMS: Root mean square; V1: Primary visual cortex; V2: Secondary visual cortex; V2M: Secondary visual cortex, medial area; VSD: Voltage-sensitive dye.

## Competing interests

The authors declare that they have no competing interests.

## Authors’ contributions

TW carried out the acquisition of VSD and ECoG data, the analysis of VSD and ECoG data and wrote the manuscript. KT carried out the acquisition of VSD and ECoG data, and participated in the analysis of VSD and ECoG data, in the preparation of the manuscript and in the design of the study. MTL carried out the acquisition of CSD data, the analysis of CSD data, and participated in the acquisition of VSD and ECoG data, and in the preparation of the manuscript. JG participated in the design of the study. FWO participated in the analysis of CSD data, in the preparation of the manuscript and in the design of the study. All authors read and approved the final manuscript.

## Supplementary Material

Additional file 1 Propagation of sensory-evoked activity does not depend on cortical state. Click here for file
