# Supplementary material for: Wave propagation of cortical population activity under urethane anesthesia is state dependent
Source: BMC Neurosci. 2013 Jul 31;14:78. doi: 10.1186/1471-2202-14-78 (PMC3733618; doi:10.1186/1471-2202-14-78)
Supplement: Additional file 1 — Propagation of sensory-evoked activity does not depend on cortical state. [file 1471-2202-14-78-S1.pdf]

Additional file 1

Propagation of sensory-evoked activity does not depend on cortical state

In order to test sensory-evoked responses, visual stimulation was provided by a white LED, positioned directly in front of the eye contralateral to the investigated hemisphere. Stimuli consisted of 30 ms full-field flashes with a fixed intensity which saturated the amplitude of the visual evoked potential. Care was taken to shield the recording light path from the visual stimulus.

As expected from previous studies (Lippert et al., 2007; Xu et al., 2007; Han et al., 2008), the spatial pattern of flash-evoked activity showed much less variability from trial to trial as compared to spontaneous activity. Each single trial response had a fixed site of initiation in V1 from where the depolarization spread across all of V1 and into V2 (Additional fig. 1A). Reflecting this fixed pattern, the calculated local propagation trajectories did not vary with cortical state within animals, nor between animals (Additional fig. 1C).

As for propagation velocities, no significant difference was found between states (Additional fig. 1B, $mean_{SYNC}$= 228 mm/s ± 24 mm/s and $mean_{DESYNC}$= 219 mm/s ± 29 mm/s (± SEM), Wilcoxon matched-pairs signed-ranks test, $p > 0.1$). However, in all six animals where sensory-evoked activity was analyzed, we found evoked waves to propagate significantly faster as compared to spontaneous waves, consistent with observations from the urethane-anesthetized auditory cortex (Sakata and Harris, 2009). On average, evoked waves propagated about twice as fast as spontaneous waves in the same cortical state (cf. Additional fig. 1B and Fig. 2D, Wilcoxon matched-pairs signed-ranks test, $p < 0.05$).

The finding that evoked activity propagates significantly faster as spontaneous activity in the same brain state contrasts with the observations of Xu et al. (2007), who found spontaneous waves in rat visual cortex to propagate faster than evoked waves. We believe the main source of this interesting discrepancy is attributable to the different anesthetic agent, with the study of Xu et al. (2007) being conducted under deep isoflurane anesthesia. Such deep

anesthesia results in a burst-suppression-like cortical activity pattern (Takagaki et al., Neurosci. Lett. 2008) which differs substantially from any of the cortical states investigated in this study. In addition, differences in the nature of the visual stimulus used (a drifting sinusoidal grating in the study of Xu et al. vs. a full-field flash in our case), as well as different computational approaches in propagation speed estimation might have further contributed to this difference.

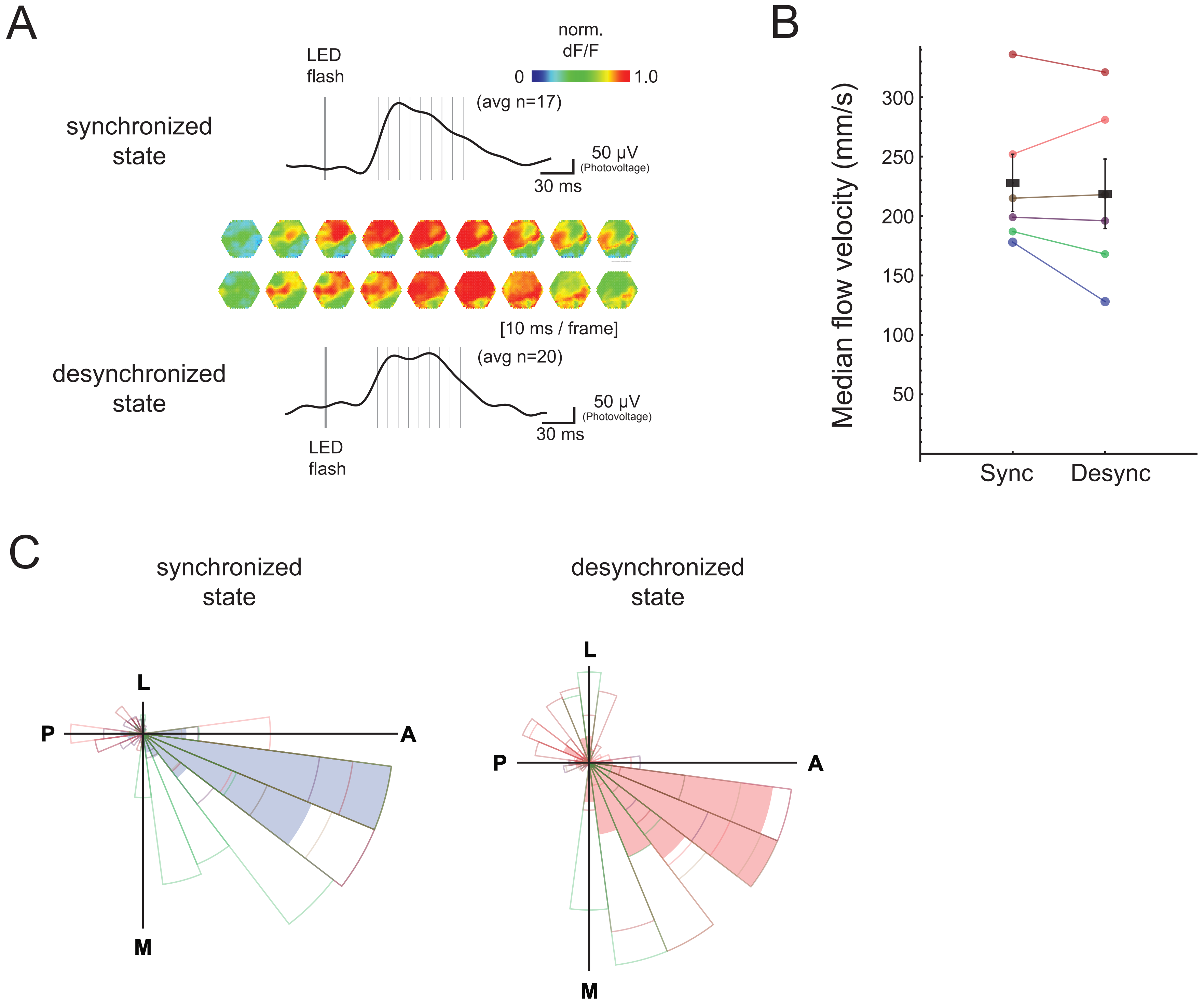
A
LED flash
norm. dF/F
0
1.0
(avg n=17)
synchronized state
50 µV (Photovoltage)
30 ms
[10 ms / frame]
(avg n=20)
desynchronized state
50 µV (Photovoltage)
30 ms
LED flash
B
Median flow velocity (mm/s)
300
250
200
150
100
50
Sync
Desync
C
synchronized state
desynchronized state
L
P
A
M
L
P
A
M

Additional figure 1. State-dependence of sensory-evoked cortical propagation. (A) Averaged visual evoked responses in a representative animal for synchronized and desynchronized states. Visual stimulation was provided by a white LED (full-field flash). Imaging field and detectors were the same as in Fig. 2. Vertical lines indicate timepoints for which frames are drawn (50 - 130 ms post-stimulus). Inset frames: frames show propagation of flash-evoked activity within the imaging field (normalized scale, variable scaling). Note that evoked activity in both states is initiated in the upper left of the imaging field corresponding to V1 and propagates along a fixed path towards the lower right (V2M). (B) Statistical evaluation of the state-dependence of evoked propagation velocity. Medians of non-Gaussian distributions from six animals for both states are shown (circles). Different colors indicate different animals, with the same color coding for animals as in Fig. 2D. For each animal, velocities were detected in single trials within a window of 200 ms poststimulus, using the same algorithm as in Fig. 2. Note that, across animals, the velocity of propagation is not considerably biased by cortical state. Means of the medians are indicated by black rectangles (± SEM). Difference of means was tested with a Wilcoxon matched-pairs signed-ranks test ($p > 0.1$). (C) Rose histograms showing propagation preferences of evoked cortical waves. Histograms were normalized to the maximum bin count for each state separately. Location of detectors was adjusted according to the site of origin of evoked activity within V1. Overlayed histograms of flow directions from six different animals are shown (colored outlines, same color coding for animals as in B). Average flow histograms (transparent blue and red) show that preferred propagation trajectories are equivalent in both states.

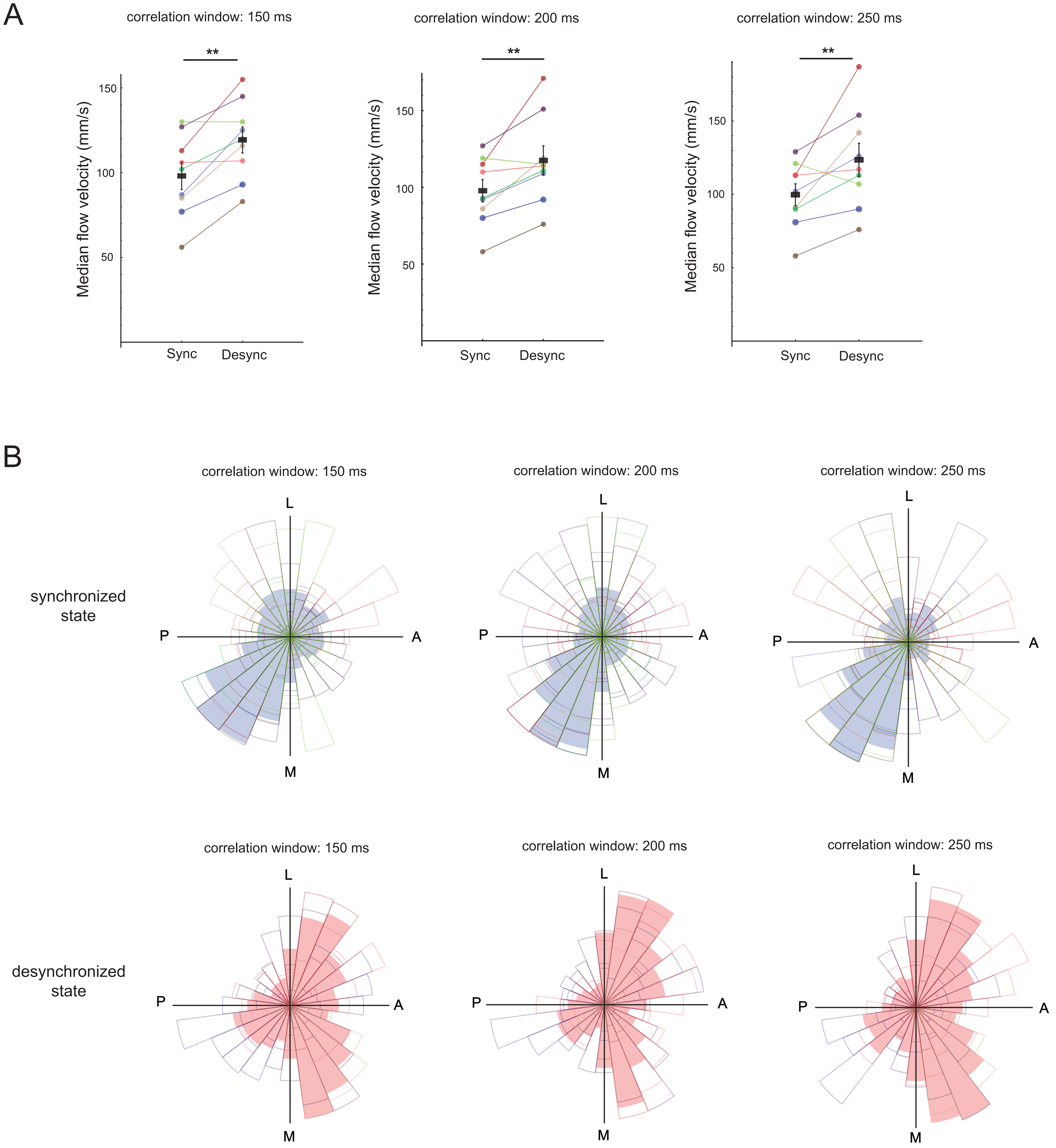
A
correlation window: 150 ms
correlation window: 200 ms
correlation window: 250 ms
**
Median flow velocity (mm/s)
150
100
50
Sync
Desync
B
synchronized state
desynchronized state
L
P
A
M

Additional figure 2. Effect of different correlation window lengths (150 ms, 200 ms, 250 ms) on flow detection. (A) Statistical evaluation of the state-dependence of propagation velocity as described in Fig. 2D. Difference of mean ranks was tested with a Wilcoxon matched-pairs signed-ranks test (**$p < 0.02$ for all three correlation windows). (B) Rose histograms showing propagation preferences of spontaneous cortical waves as described in Fig. 2E. Note that for the desynchronized state, overlayed histograms of flow directions are shown from five animals, as compared to nine animals in Fig. 2E, which resulted in a stronger weighting of the average flow in anterior-medial direction. The exclusion of four animals from the average in this rose histogram was due to the lower sensitivity of flow detection with a 150 ms correlation window (mean value of flow events detected per animal: 1750 events for a 150 ms window, 2635 for a 200 ms window and 2902 for a 250 ms window), which resulted in these four animals falling below the minimum criterion for wave events detected per animal, set at 100 events per animal. However, note that neither the propagation bias for the lateral-medial axis, nor the state-dependence of preferred propagation directions within this biased axis (Kuiper's test, $p < 0.001$ for every animal) were affected by the correlation window length.
